# Quail eggs in artificial nests change their coloration when exposed to ambient conditions: implication for studies on nest predation

**DOI:** 10.7717/peerj.11725

**Published:** 2021-07-06

**Authors:** Gustavo Liñan Cembrano, Macarena Castro, Juan A. Amat, Alejandro Perez, Miguel Ángel Rendón, Cristina Ramo

**Affiliations:** 1 Instituto de Microelectrónica de Sevilla (IMSE-CNM-CSIC—Universidad de Sevilla), Seville, Spain; 2 Instituto Universitario de Investigación Marina (INMAR), Campus de Excelencia Internacional del Mar (CEIMAR), Universidad de Cádiz, Puerto Real, Cádiz, Spain; 3 Dpto. Ecología de Humedales, Estación Biológica de Doñana (EBD-CSIC), Sevilla, Spain

**Keywords:** Artificial nests, Egg coloration, Japanese Quail, Nest camouflage, Open nests, Predation studies

## Abstract

Quail eggs have been widely used in field experiments, mainly to study factors associated with the risk of nest predation. Some shortcomings of using quail eggs in this type of study have been previously addressed (e.g., these eggs might be too big for some predators of eggs of small birds). Here, we show experimental evidence of another shortcoming of the use of these eggs in field experiments. Quail eggs exposed to sunlight rapidly faded in colour after three days, both in the visible and UV spectra, and this change was related to the amount of solar radiation received. This caused changes in the camouflage of the eggs, which may be perceived by predators with different visual systems (dichromatic, trichromatic, and tetrachromatic (for both violet- and UV-sensitive species)). Therefore, the results of field studies of nest predation using quail eggs might be questioned in those cases in which the camouflage has been altered due to the rapid changes in coloration, as this can affect the resulting predation rates. We recommend that researchers planning to use quail eggs should perform a prospective assessment of changes in coloration of eggs exposed to environmental conditions in the nest sites used by the target species.

## Introduction

Experimentation is required to explain the processes and patterns observed in nature, and, for this, an understanding of the methods of the experimental design is very important (James & McCulloch, 1985). Eggs of Japanese Quail *Coturnix japonica* have been frequently employed in experimental studies of avian nesting ecology, including camouflage (Lovell et al., 2013), thermal environment (Gómez et al., 2016), nest parasitism (Yang et al., 2017), and mainly nest predation (Huhta, Jokimäki & Helle, 1998; Nguyen, Abraham & Nol, 2007; Zuria, Gates & Castellanos, 2007; Rocha et al., 2016; Ferreira-Rodríguez & Pombal, 2018; Bravo et al., 2020). Although in some situations the predation of artificial nests may represent an adequate measure of real predation (Hoset & Husby, 2019), in other cases it might not represent adequately what occurs under natural conditions, because the predation of artificial nests may differ from that of real nests (Moore & Robinson, 2004). This may be because artificial nests are not subject to the same conditions as real nests (e.g., size of eggs, lack of parental care) and may attract different species of predators (Major & Kendal, 1996; Moore & Robinson, 2004).

One widely accepted shortcoming of using quail eggs in studies of nest predation is that these eggs might be too big for some small egg predators (Roper, 1992; Lindell, 2000; but see Craig, 1998; Lewis & Montevecchi, 1999). We had previously used commercial quail eggs during field studies on Kentish Plover *Charadrius alexandrinus* nesting success at two sites and in 3 years in southern Spain and noticed that the coloration of these eggs changed after only a few days of exposure to ambient conditions. Given the importance of egg camouflage for nesting success (Skrade & Dinsmore, 2013; Troscianko et al., 2016), the changes in coloration that quail eggs experience when directly exposed to ambient conditions may represent another shortcoming of using this type of eggs in field studies of nest predation, since these changes in coloration may affect their camouflage, and likely predation rates. Motivated by this, we performed two field experiments to quantify the temporal changes in coloration that quail eggs experience when exposed to ambient conditions and measured how this could affect the camouflage of eggs. This may be especially relevant for studies in which the target species is of conservation concern, as biased results based on the experimental use of quail eggs to assess nest predation rates of such species may lead to erroneous management decisions.

## Material & methods

Our study was conducted in 2020 at a saltpan in Cádiz Bay Natural Park, southern Spain (36° 30′ 53.4″ N, 6° 09′ 23.3″ W). We performed two experiments, one focused on the effect of solar radiation on the colour of the eggs, and another one to quantify the effect of the change in coloration on egg camouflage.

### Experiment 1

We used 50 commercial eggs of Japanese Quail and placed them under field conditions in five hand-made scrapes at ground level (hereafter simulated nests), and used different levels of shading to make the eggs to receive different amounts of solar radiation, thus simulating nests with different levels of vegetation cover. We deposited 10 eggs on the ground within a hand-made scrape with a diameter of about 15 cm, in three rows of three, four and three eggs, respectively (Supplemental Information, Fig. S1). These 10 eggs were deposited in a site exposed to direct sunlight and were protected with a cylinder of metal mesh to minimize the risk of predation. The remaining eggs were gradually shaded as follows: 10 with one layer of white cloth firmly attached to the metal mesh, 10 with two layers of white cloth, 10 with four layers of white cloth, and 10 with one layer of black cloth. The white cloth is a non-woven fabric whose average reflectance (measured in a circle of 2.5cm of diameter) is 49.7% while the black cloth is cotton and its average reflectance is 5.9% (measured in a circle of 2.5cm of diameter).The distance between simulated nests was about 50 cm (Fig. S1).

To measure the differences in the solar radiation received on each of the five simulated nests, we added one Odroid Weather 2 sensor (https://www.hardkernel.com/shop/weather-board-2/) to each one. We placed the sensors in a similar position and orientation with respect to the north within each nest, and 20 cm above the ground. We connected each sensor to a Single Board Computer Odroid C2 (https://www.hardkernel.com/shop/odroid-c2/) powered by a 5V 10,000 mAh battery embedding a Real-Time Clock Odroid RTC Shield (https://www.hardkernel.com/shop/rtc-shield/) that allowed for the synchronization of the five systems. We programmed the systems to monitor UV and Visible irradiance conditions. Information about UV conditions was stored as the existing UV index, as established by World Health Organization (World Health Organization, 2002). Irradiance in the visible wavelength range was recorded in *lux* (the derived SI unit of illumination, equal to 1 lumen per square meter), and also in watts per square meter to compare to Hutchinson & Matt (1977) luminance levels. The system was programmed to sample this information from the sensors every 5 min during 12 hours (starting at 10:00 h, GMT) and to save the information in USB memory sticks. Environmental data recording was performed on the first day of the experiment and repeated on days 3 and 7 to account for the possible different rates of degradation of the covering cloths over time. For each simulated nest, the maximum radiation measurements were averaged for the three days.

All eggs were from different females, as judged by different eggshell spotting patterns (see Gosler, Barnett & Reynolds, 2000; Skrade & Dinsmore, 2013), which precluded that there was pseudoreplication. Since we were simply interested in analysing the effects of solar radiation on eggshell coloration, by placing all eggs in a single site we avoided the effects of any confounding factors that could be associated to specific sites.

We took pictures of eggs at the beginning of the experiment, three days later (day 3), and one week later (day 7). Initially, we had planned to take pictures also in day 14, but Yellow-legged Gulls *Larus michahellis* removed the protections after a week and depredated all the eggs. Pictures were taken both in the visible range (VIS, 400–700 nm) and in a region (350–400 nm) of the ultraviolet band (inside the UVA range 315–400 nm) using a UV-converted Sony ILCE 6000 camera equipped with a Sony 18–55 mm lens, and mounted on a tripod. We used a delayed trigger to suppress blurring effects in the long term UV exposures due to manual operation. For the VIS images, we used a Fotga UV-IR cut filter, whereas for the UV images we employed a Baader U UV-pass filter. In each photo session, we removed the cover of each nest, placed a UV and VIS grey standard (SphereOptics, 25% of reflectance) next to the eggs and obtained two images (VIS and UV). Pictures (RAW) were taken zenithally at f/8 and at a height of approximately 50 cm. The digital images were linearized and normalized to the grey standard to obtain reflectances (Troscianko & Stevens, 2015; Gómez & Liñán-Cembrano, 2017). The image processing, for both UV and VIS sets, was executed using the tool SpotEgg (Gómez & Liñán-Cembrano, 2017) that allowed to obtain equivalent reflectance images from the RAW files and to draw regions of interest (RoIs) as closed polygonal lines around each egg. As measures of reflectance (color), we used the mean values of the three camera bands (red, green and blue) in the VIS, and in the UV spectra.

### Experiment 2

To quantify how eggs’ camouflage may be affected by changes in coloration resulting from their exposition to ambient conditions, we placed quail eggs in 15 nests (3 eggs per nest) that had been previously used by Kentish Plovers during the breeding season of 2020. These eggs were from different females. We took pictures of these nests at the beginning of the experiment, 1 week later (day 7) and 2 weeks later (day 14); however, only 7 nests had eggs at day 14, while 7 nests were depredated between days 1 and 7, and one more between days 7 and 14. The pictures were taken at a distance of 1 m, using the same camera and settings as in the first experiment. We processed the images (linearization, normalization, definition of RoIs) using SpotEgg (Gómez & Liñán-Cembrano, 2017), obtained reflectance images and extracted the reflectance in the selected RoIs (eggs).

To evaluate changes in camouflage in the artificial nests along time, we ran a texture analysis (see Gómez et al. (2019) for details). Texture can be described by the number and types of texture elements (e.g., structural, spectral, fractal), and by their relative spatial relationships, a process that is called texture classification (Hunt & Pointer, 2011). The use of texture analysis to evaluate the degree of camouflage is well stablished in the literature (see Nyberg & Bohman, 2001; Martin, Fowlkes & Malik, 2004). In our analysis we computed the texture content on each image by convolving each of the channels in the image with a set of 14 different filters defined at 4 scales (σ = {16, 32, 64, 128} pixels). The filter set at each scale contains odd-symmetric and even-symmetric Gaussian derivative kernels at five orientations (evenly distributed between 0 and 180°), plus four rotation invariant filters (two Gaussian kernels, two Laplacian of Gaussian kernels). Thus, for instance, for a three channels colour image we produced 168 (3 × 56) versions of the input image. These measurements were vectorized and clustered using K-means for 20 cluster centres. Each cluster centre defines the position of texture in the, for instance, 168 variables hyper-space that is created for a three-channel colour image. Finally, we created the output image by computing the Euclidean distance between the measurements for a given pixel (168 in the example) and the position of the defined (20) cluster centres and labelled every pixel in the output image as belonging to the closest texture centre. Then, we created the histograms of the texture contents, what can be understood as a texture signature. In other words, we counted how many pixels were present in a region for every texture label. We ran this calculation for the eggs area, and the microhabitat, a 45 cm diameter circle, surrounding the nest region. We normalized the histograms so that each bin represents the number of pixels assigned to a texture divided by the total number of pixels in this area, and we computed the difference between the eggs area and microhabitat region normalized histograms using the χ^2^ metrics defined in Arbeláez et al. (2011). Briefly, the lower the difference between the distribution of textures in the two regions, the more similar the regions.

Since our goal was to assess whether coloration changes in quail eggs may affect their detection by predators, the images subject to texture analysis were not the images obtained in the field but the result of mapping these images to the visual models of predators with four visual systems: dichromatic (Ferret *Mustela putorius*, with sensivity peaks at [430, 538] nm) (Calderone & Jacobs, 2003), trichromatic (human, with peaks at [420, 533, 562] nm) (Bowmaker & Dartnall, 1980), tetrachromatic violet-sensitive (Peafowl *Pavo cristatus* with peaks at [421, 457, 505, 566] nm) (Hart, 2001), and tetrachromatic ultraviolet-sensitive (Blue Tit *Cyanistes caeruleus* with peaks at [371, 448, 503, 563] nm) (Hart, 2001). The mathematical description of the process behind the creation of these visual-models mapped images is summarized in File S1 and Fig. S2 for illustration purposes.

Field experiments conducted at Parque Natural Bahia de Cádiz, Consejería de Medio Ambiente, Junta de Andalucía, with explicit permission from its Director. Antonio Gómez Ferrer.

### Statistical analyses

Generalized linear mixed model (GLMM) with restricted maximum likelihood (package nlme, Pinheiro et al., 2018) was used to test differences in reflectance in two spectra (VIS, UV), three moments (day 0, day 3 and day 7) and five treatments (exposed, and shaded with a white layer, two white layers, four white layers, and a black layer), with egg identity as random factor. The response variable (egg reflectance) was arcsine square-root transformed to meet the requirement of normality and homoscedasticity. By using a mixed model, we avoid any possible pseudoreplication problem due to repeated-measures design (Zuur & Ieno, 2016). We used Tukey post-hoc to assess differences between treatments (package emmeans, Lenth et al., 2019). Also using a GLMM, we analysed the reflectance of the eggs placed in Kentish Plover nests in two spectra (VIS and UV) and three moments: day 0, day 7 and day 14, with the nest as random factor, checking the normality of the residuals (through visual inspection) and the homogeneity of variances (Levene’s Test).

We used Pearson correlations to compare the solar radiation measurements in each simulated nest and the average change of reflectance of the eggs in each simulated nest after 7 days, both in VIS and in UV (package MASS, Ripley et al., 2019).

We also used GLMM to examine variations in camouflage in three moments (day 0, day 7 and day 14). The response variable was the differences in textures between eggs and microhabitat surrounding nests, the independent variable was the day (0, 7 and 14) and the random factor, the nest.

All statistical analyses were carried out using R statistical software version 3.5.0 (R Core Team, 2018) and the significance level was set at 0.05.

## Results

**Figure 1:**
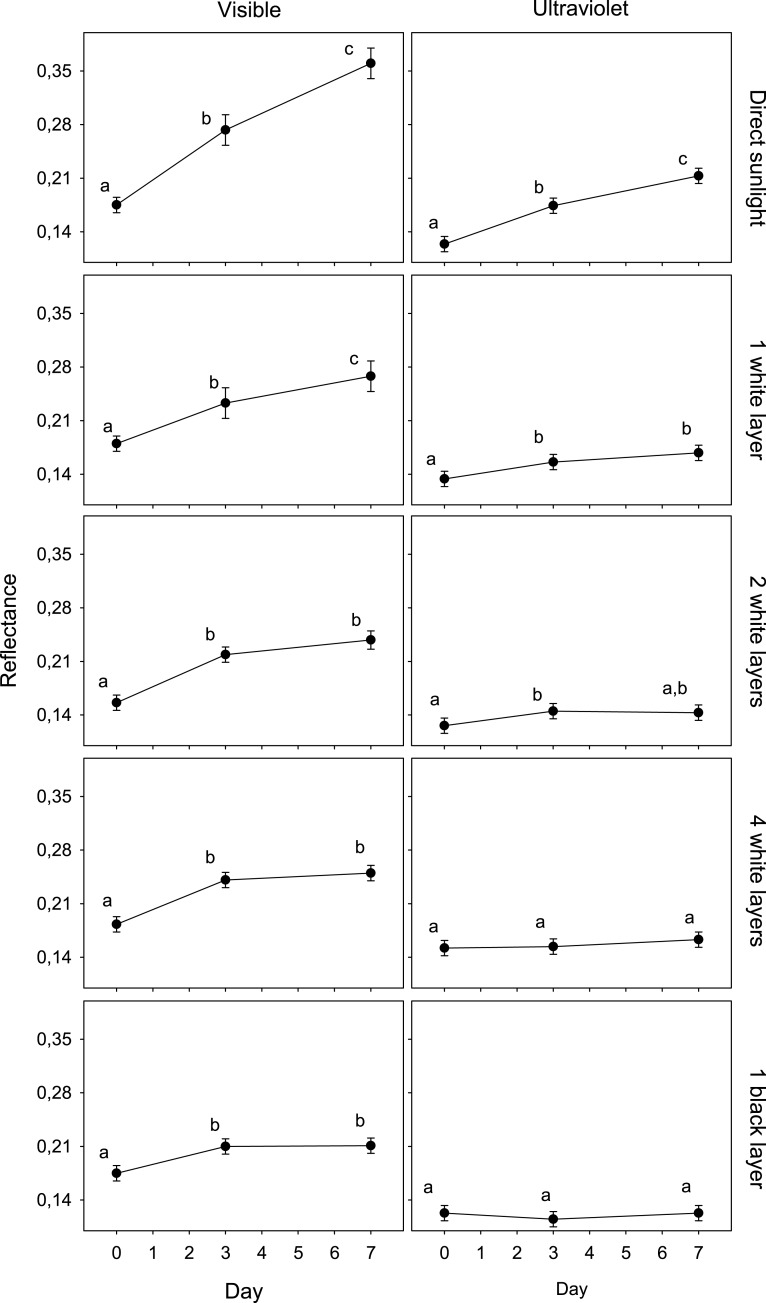
Variation in egg reflectance under five treatments over 7 days. Changes in visible (VIS) and ultraviolet (UV) reflectance (means ± SE) across time of Japanese Quail eggs undergoing five treatments (10 eggs per treatment). Generalized linear mixed models were used to test differences in reflectance (arcsine square-root transformed) in three times since exposure (days 0, 3 and 7) and the five treatments. Different letters denote significant differences in the reflectance of eggs (Tukey post-hoc comparisons).

The results of the GLMM of the first experiment showed an interaction between spectrum, day and treatment (*F*_8,225_ = 3.5; *P* < 0.001; Table 1). The reflectance of eggshells in VIS changed significantly after only 3 days in all treatments, and again after 4 more days in the case of exposed and shaded eggs with one white layer. In UV, this value only changed in the cases of eggs exposed to direct sunlight and shaded with one and two white layer after 3 days (Fig. 1).

**Table 1:** Results of the GLMM in the first experiment. A generalized linear mixed model was used to test differences in quail egg reflectances in two spectra (VIS, UV), three times since exposure (day 0, day 3 and day 7) and five treatments (exposed, and shaded with a white layer, two white layers, four white layers, and a black layer of cloth), with egg identity as random factor. The response variable (egg reflectance) was arcsine square-root transformed to meet the requirement of normality and homoscedasticity. See text for the characteristics of both white and black cloth.

	DF	*F* value	*P* value
Intercept	1/225	612.7	<0.0001
Day	2/225	141.4	<0.0001
Treatment	4/45	1.5	0.2098
Spectrum	1/225	101.4	<0.0001
Day:Treatment	8/225	23.3	<0.0001
Day:Spectrum	2/225	43.0	<0.0001
Treatment:Spectrum	4/225	4.4	0.0020
Day:Treatment:Spectrum	8/225	3.5	0.0009

The reflectance of eggs exposed to ambient conditions varied from an average of 0.175 (day 0) to 0.361 (day 7) (an increase of 106.1%) and 0.124 (day 0) to 0.213 (day 7) (an increase of 71.4%) in the visible and ultraviolet spectra, respectively. The reflectance of eggs covered with a black coat varied from 0.175 to 0.211 (an increase of 20,3%) and 0.123 to 0.123 (0.1%) in the visible and ultraviolet spectra, respectively. The other treatments presented intermediate values. These increases were significantly correlated with the solar radiation recordings (VIS, UV) in each treatment (Table 2; *n* = 5; all *r* > 0.98; all *P* < 0.05).

**Table 2:** Egg reflectance increases and radiation received in five treatments. Increases in egg reflectance (expressed as average percentages of final values with respect to initial values, Illuminance (W/m^2^) and UV-index) of eggs of Japanese Quail after a week of exposure to ambient conditions. In the treatments the eggs received direct solar radiation or were covered with different layers of cloth. See text for the characteristics of both white and black cloth.

	∆ Egg reflectance		Received radiation	
Treatment	∆ VIS (%)	∆ UV (%)	Illum. (W/m^2^)	UV-index
Direct sunlight	106.07	71.44	730	7.5
1 white layer	48.64	25.56	224	2.3
2 white layers	52.78	13.55	160	1.6
4 white layers	37.08	7.38	97	1.0
1 black layer	20.26	0.14	7	0.1

**Figure 2:**
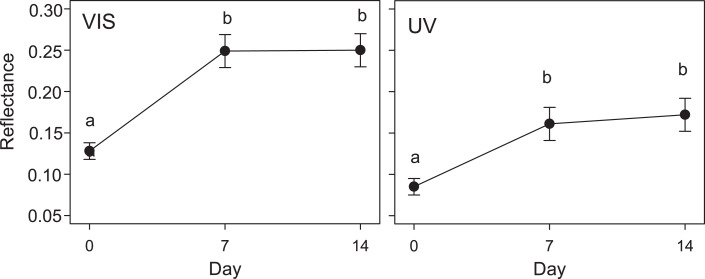
Variation in quail egg reflectances placed in Kentish plover nests over 14 days. Changes in visible (VIS) and ultraviolet (UV) reflectance (means ± SE) across time of Japanese Quail eggs placed in 7 Kentish Plover nests (3 eggs per nest). Generalized linear mixed models were used to test differences in reflectance in three times since exposure (days 0, 7 and 14). Different letters denote significant differences (Tukey post-hoc comparisons).

**Figure 3:**
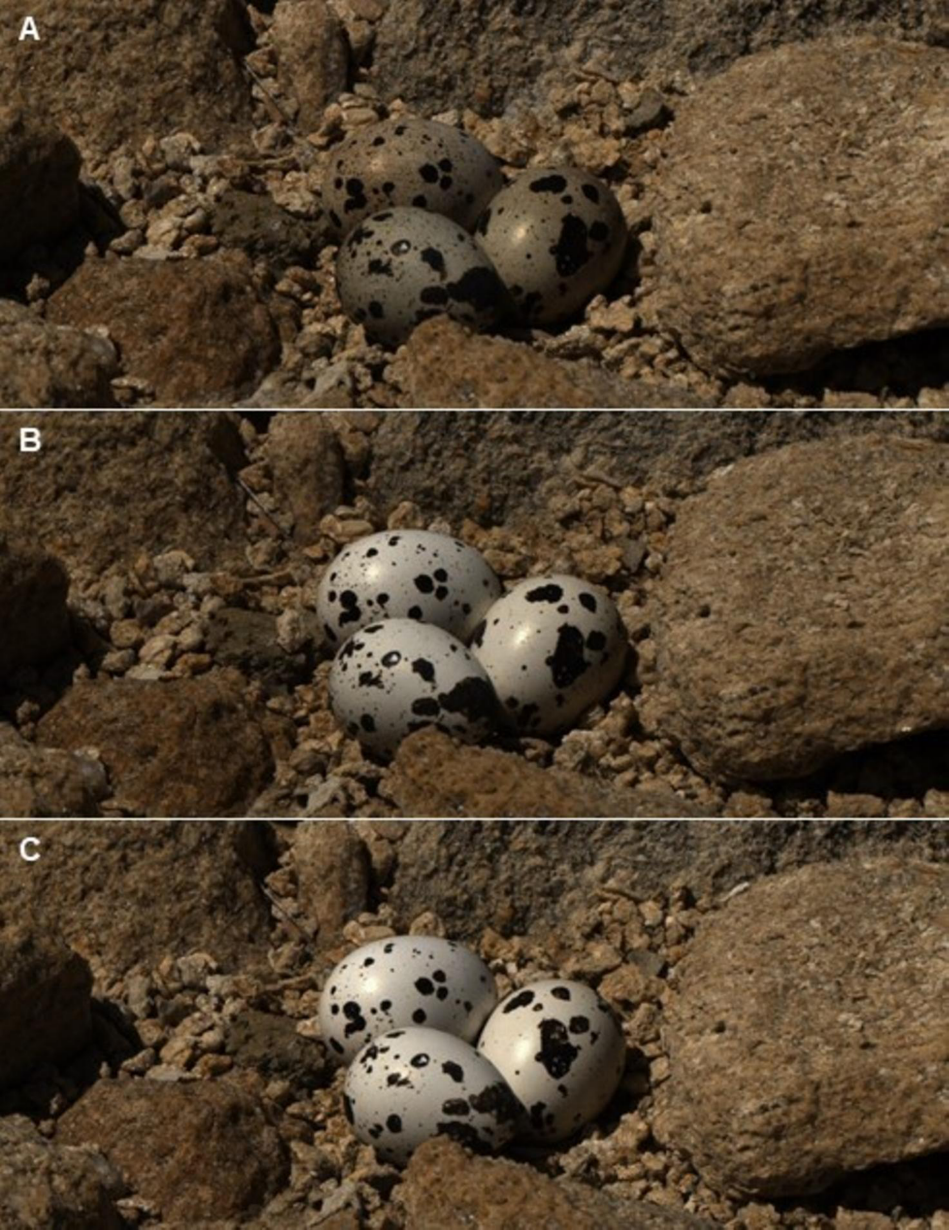
Reflectance images of quail eggs. Reflectance images (after linearization and normalization) of three quail eggs placed in a Kentish plover nest at the initial exposure to ambient condition (A), after 7 days (B) and 14 days (C).

The results of the GLMM in the second experiment were in line with those of the first experiment: the reflectance of eggs placed in Kentish Plover nests (i.e., receiving direct solar radiation) increased significantly after a week, both in VIS and UV (*F*_2,30_ = 3.6; *P* < 0.05) (Figs. 2 and 3). A consequence of this was that the camouflage of eggs with respect to the microhabitat surrounding the nests worsened significantly after a week according to the four visual models considered (Table 3).

**Table 3:** Variation in egg camouflage over 14 days. Differences in camouflage (means ± SE) of Japanese Quail eggs with respect to the microhabitat around Kentish Plover nests (*n* = 7), calculated with an analysis of textures, and according to four visual models, in the initial day (Day 0) and after 7 (Day 7) and 14 (Day 14) days of exposure to ambient conditions. Lower values represent better camouflage. Generalized linear mixed models, with nest identity as random factor, were used to test differences between three times since exposure (days 0, 7 and 14). Different letters denote significant statistical differences between days (Tukey post-hoc comparisons. *P < 0.05, **P < 0.005).

Visual model	Day 0	Day 7	Day 14	*F*_(2.12)_
Dichromatic (Ferret)	6.90 ± 0.44^a^	9.09 ± 0.75^b^	9.39 ± 0.65^b^	5.83*
Trichromatic (Human)	6.98 ± 0.43^a^	9.18 ± 0.65^b^	9.36 ± 0.03^b^	7.01**
Tetrachromatic, V-sensitive (Peafowl)	7.00 ± 0.45^a^	9.12 ± 0.83^b^	9.51 ± 0.88^b^	5.42*
Tetrachromatic, UV-sensitive (Blue Tit)	7.34 ± 0.50^a^	9.42 ± 0.81^b^	9.45 ± 0.59^b^	4.45*

## Discussion

An important requirement of any experimental approach is that it should reproduce adequately the natural conditions experienced by the factor under analysis. If this requirement is not met, the results of the experiment may be flawed. Quail eggs in artificial nests are continuously exposed to direct ambient conditions, in contrast to eggs in natural nests that usually are covered by the incubating parents and only remain exposed during short periods. Here, we have shown that (1) the coloration of quail eggs exposed to sunlight changed after a very few days, both in the VIS and UV wavelength ranges, (2) this change was related to the amount of solar radiation received, and (3) this change in coloration affected camouflage.

In studies where artificial nests with quail eggs are used, researchers usually take care to place them in sites that resemble those where natural nests are found. When the sites are partially covered by vegetation or rocks, experimental quail eggs could not receive so much solar radiation as the eggs in completely exposed sites, although eggs in partially covered sites could still receive enough solar radiation as to change their colour.

There are some studies that report solar radiation that might impact organisms on the ground as well as through the canopy, such as a study from Tennessee, U.S.A. (maximum total radiation recorded on 14th June 1972, at midday; Hutchinson & Matt, 1977), conducted at a similar latitude to that of our study area (36° 00′ 37″ N, 84° 16′ 11″ W), showed that the maximum radiation received above the forest canopy (1,014 W/m^2^) was even higher than that received in our treatment of direct solar radiation (730 W/m^2^). The radiation received in the upper canopy (343 W/m^2^) was also higher than that received in the treatment with 1 white layer (224 W/m^2^). The radiation received by the treatment with 2 white layers (160 W/m^2^) was similar to that received in the mid-canopy (184 W/m^2^), and the radiation received in the treatment with 4 white layers (97 W/m^2^) was very similar to that received at ground level in the forest (102 W/m^2^). Therefore, it may be expected that in the nests that occupy these positions in a forest, the eggs would fade in a similar way than those of our treatments.

The changes in eggshell coloration experienced by quail eggs would render the eggs more easily detectable by predators with different visual capabilities (non UV- (either dichromatic or trichromatic), violet- and UV-sensitive). In ground nests that are exposed, egg coloration must match the surrounding microhabitat, because well camouflaged clutches have higher survival probability than those in which camouflage is worse (Skrade & Dinsmore, 2013; Troscianko et al., 2016). Some studies using quail eggs found differences in predation rates of eggs in artificial nests according to nest cover (Chibowski, Brzeziński & Jedlikowski, 2015; Arbeiter & Franke, 2018) but did not analyze whether there were changes in eggshell coloration that could have affected the resulting predation rates. Rangen, Clark & Hobson (2000) addressed some limitations of the use of artificial nests in studies of avian nest predation and recommended using quail eggs when predator assemblages are dominated by large predators.

In addition to nest predation studies, quail eggs have also been used in studies of nest parasitism (Yang et al., 2017), and changes in quail eggshell coloration could affect mimicry (Hanley et al., 2016).

Given our results, we suggest that researchers planning to use quail eggs should perform a prospective assessment of changes in coloration of eggs exposed to environmental conditions in the nest sites used by the target species, especially when the main predators locate the nests by sight.

## Conclusions

Quail eggs are usually left in artificial nests for at least 7 days (e.g., Huhta, Jokimäki & Helle, 1998; Zuria, Gates & Castellanos, 2007; Rocha et al., 2016; Ferreira-Rodríguez & Pombal, 2018; Bravo et al., 2020), and we have shown that the eggs fade in coloration during such period, so that the results of studies of nest predation using quail eggs might be questioned, especially in the case of open nests exposed to direct sunlight, as their camouflage may change, and this could affect predation rates.

## Supplemental Information

10.7717/peerj.11725/supp-1 Supplemental Information 1 Visual-models mapped images and Sample Figures. Mathematical description of the process to generate visual-model mapped images together with complementary information pictures.

10.7717/peerj.11725/supp-2 Supplemental Information 2 Raw experimental results for the two experiments reported in the article.

## Additional Information and Declarations

### Field Study Permissions

The following information was supplied relating to field study approvals (i.e., approving body and any reference numbers): Field experiments conducted at Parque Natural Bahia de Cádiz, Consejería de Medio Ambiente, Junta de Andalucía, with explicit permission from its Director. Antonio Gómez Ferrer.

### Data Availability

The following information was supplied regarding data availability: The raw experimental measurements are available in the Supplemental File.
