# Supplementary material for: Quail eggs in artificial nests change their coloration when exposed to ambient conditions: implication for studies on nest predation
Source: PeerJ. 2021 Jul 6;9:e11725. doi: 10.7717/peerj.11725 (PMC8269738; doi:10.7717/peerj.11725)

**Supplemental information**

**File S1. Visual-models mapped images: Mathematical description of the process.**

First, we employed our optical test-bench to measure the spectral response of the different colour channels of the camera when interposing the Fotga UV-IR cut filter and the Baader U UV-pass filter. In short, light from a Newport 66884 lamp was directed through an Oriel USFW-100 filter wheel (to suppress high order back reflected harmonics in the monochromator) into a Newport CS130 monochromator which was programmed to execute wavelength sweeps in the range [350, 700] nm with 5nm steps. The monochromator output beam was directed to a SphereOptics SPH-2Z integrating sphere whose output beam was passed to a 50/50 OFR beam splitter. The beam splitter balanced output beams were finally directed into this experiment's camera and a calibrated light power meter respectively. Such setup allowed us to measure camera response vs. wavelength for the different color channels (R, G, and B) when using the UV-IR cut filter and the UV-pass filter. Second, we employed a parameterized expression for the absorbance spectra of rhodopsins (Govardovskii 2000) and the information about the wavelength peaks for the selected animals to obtain the spectral sensitivity curves associated to each model predator colour vision. Afterwards, we simulated the response of the camera (VIS and UV) and that of the animal visual models using the CIE D65 standard (CIE 1986) as illuminant and different (nearly 720,000) object reflection spectra, namely: the 1600 glossy Munsell color chips available in Spectral Database (https://www3.uef.fi/es/web/spectral/-spectral-database), 218 natural colours reflectance spectra from (Parkkinen 1998), 1056 forest colours from (Jaaskelainen 1994) and approx. 715,000 artificially created non-flurorescent smooth spectra in (Flinkman 2012). Finally, we trained a 16×32×32×8×N (being N the number of colour channels in the model animal) feedforward neural network for each visual

model to map the camera simulated outputs to the visual model simulated outputs. Therefore, this network's input during training was the result of simulating the camera sensors responses for these combinations of measured colour sensitivity curves, D65 illuminant, and object reflectances in the databases above and as outputs to be fitted the results of simulating the visual model outputs for the cone sensitivity curves and the same combination of illuminant and object reflection spectra. The training of the networks converged, with the correlations coefficients between simulated outputs and neural networks outputs being $r > 0.9999$ in all cases. All the calculations and neural network training were executed in MATLAB R2020a and the fitted networks stored as MATLAB structures in a separated file for each visual model. We then passed the reflectance images obtained with SpotEgg (Gómez and Liñán-Cembrano 2017) to the fitted neural networks to obtain the simulated visual-model images and used these images as inputs during our texture analysis (Gómez et al. 2019) (see Fig. A2 for illustration purposes).

CIE Colorimetric illuminants, 1986. ISO/CIE 10526 – 1991 (CIE S001-1986). http://cie.co.at/publications/colorimetry-part-2-cie-standard-illuminants. Last accessed Sept. 2020

Flinkman, M., Laamanen, H., Vahimaa, P. and Hauta-Kasari, M. 2012. Number of colors generated by smooth nonfluorescent reflectance spectra. – J. Opt. Soc. Am. A 29: 2566-2575. https://doi.org/10.1364/JOSAA.29.002566

Gómez, J. and Liñán-Cembrano, G. 2017. SpotEgg: An image-processing tool for automatised analysis of colouration and spottiness. – J. Avian Biol. 48: 502-512. https://doi.org/10.1111/jav.01117

Gómez, J., Liñán-Cembrano, G., Ramo, C., Castro, M., Pérez-Hurtado, A. and Amat, J. A. 2019. Does the use of nest materials in a ground-nesting bird result from a compromise between the risk of egg overheating and camouflage? – Biol. Open 8: bio042648. https://doi.org/10.1242/bio.042648

Govardovskii, V. I., Fyhrquist, N., Reuter, T., Kuzmin, D. G. and Donner, K. 2000. In search of the visual pigment template. – Vis. Neurosci. 17: 509–528. https://doi.org/10.1017/S0952523800174036

Jaaskelainen, T., Silvennoinen, R.,Hiltunen, J. and Parkkinen, J. P. S. 1994. Classification of the reflectance spectra of pine, spruce, and birch. – App. Op. 33: 2356–2362.

Parkkinen, J., Jaaskelainen, T. and Kuittinen, M. 1988. Spectral representation of color images, ' IEEE 9th International Conference on Pattern Recognition, Rome, Italy, 14–17 November, 1988, Vol. 2, pp. 933–935.

**FIGURE S1**. Five simulated nests, each one containing 10 Japanese Quail eggs, used in the experiment. Each nest was provided with a sensor to measure radiation. The nests were protected with a cylinder of mesh to avoid predation.

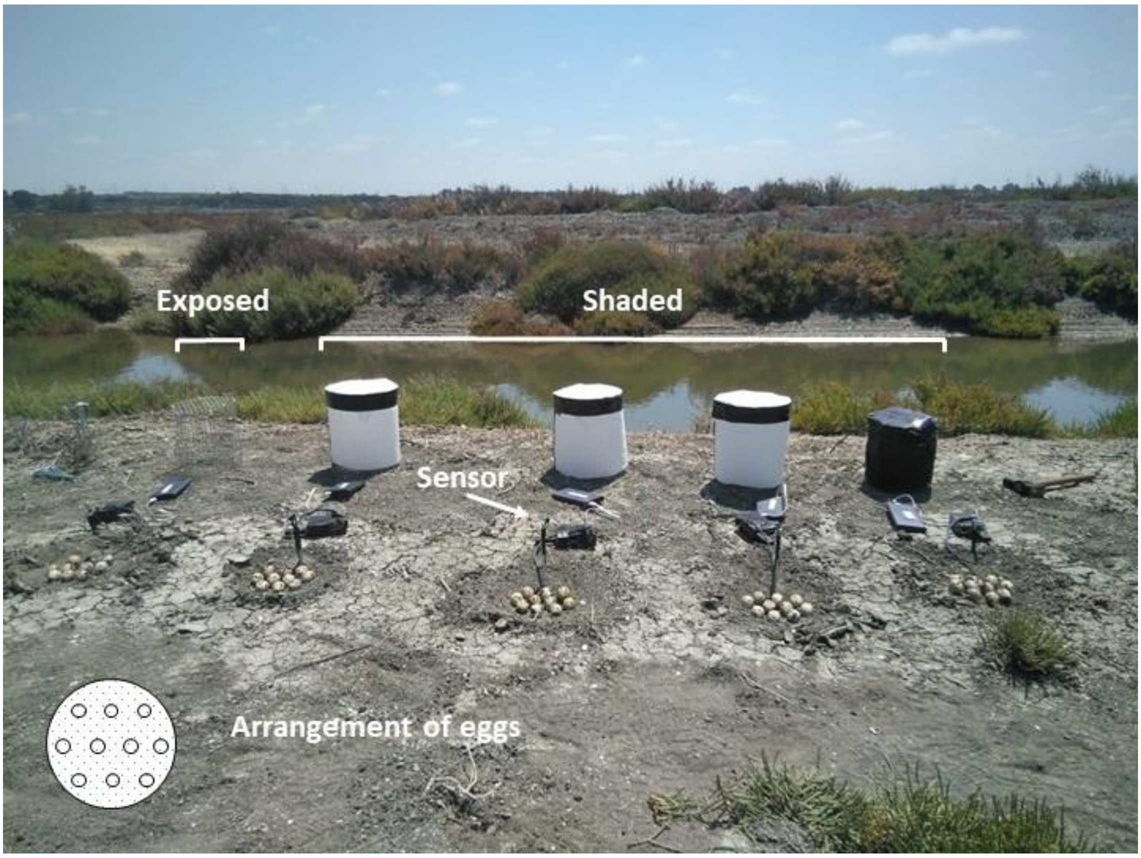

**FIGURE S2**. Input images and texture images in experiment 2. (a.1) UV reflectance image, (a.2) VIS reflectance image, (b.1) texture image for the Peafowl simulated visual model, (b.2) texture image for the Blue Tit simulated visual model, (b.3) texture image for the human simulated visual model, and (b.4) texture image for the Ferret simulated visual model.

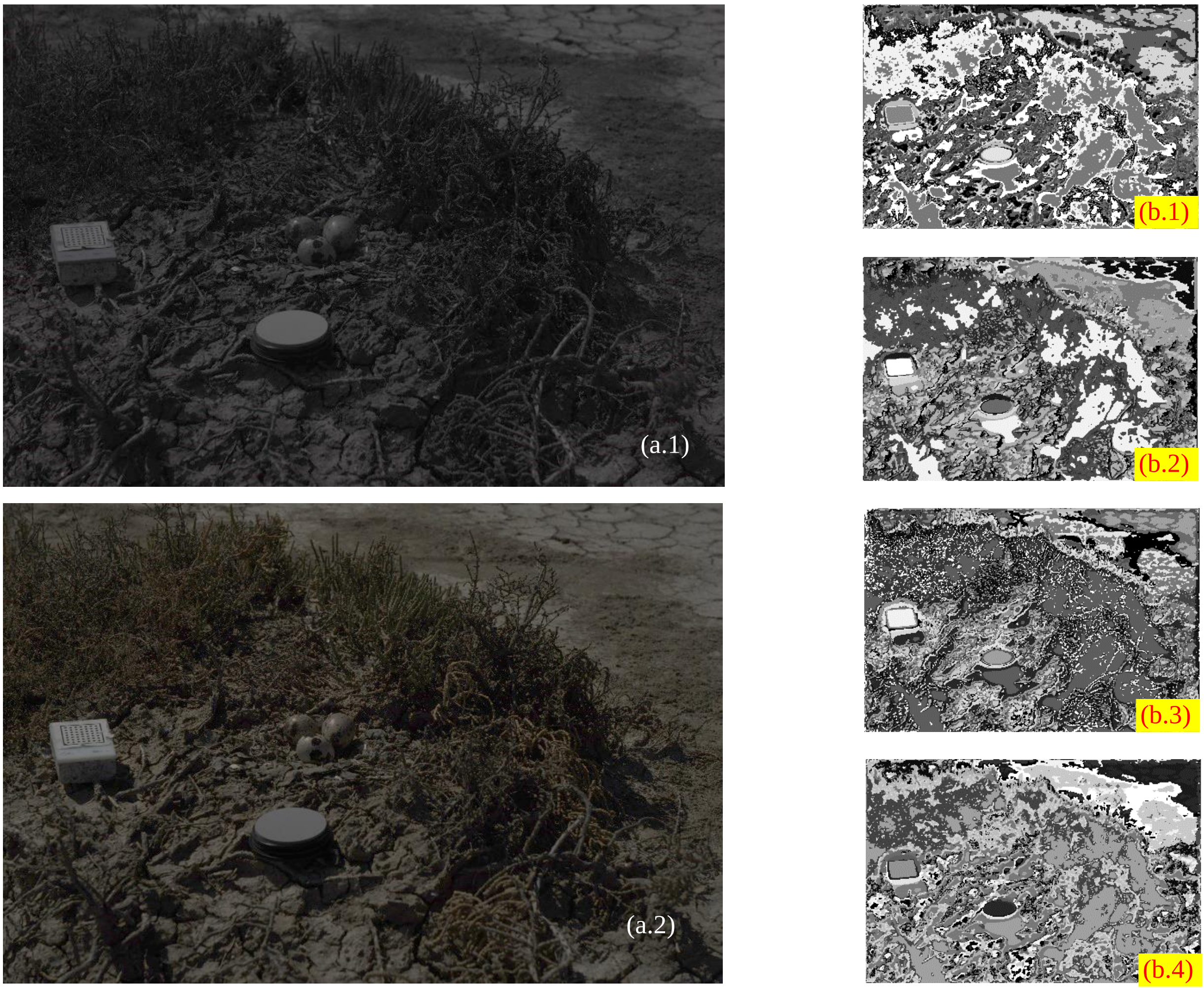

Supplement: Supplemental Information 1 — Mathematical description of the process to generate visual-model mapped images together with complementary information pictures. [file peerj-09-11725-s001.pdf]
